# Strength of associations of psoriatic arthritis and physical activity with body composition: the population-based Trøndelag Health study

**DOI:** 10.1007/s10067-026-08252-2

**Published:** 2026-06-25

**Authors:** Abdirizak Ali Osman, Mari Hoff, Paul Jarle Mork, Vibeke Videm

**Affiliations:** 1 Department of Clinical and Molecular Medicine, NTNU - Norwegian University of Science and Technology, St. Olavs University Hospital, Lab Center 3 East, NO-7006 Trondheim, Norway; 2 Department of Neuromedicine and Movement Science, NTNU - Norwegian University of Science and Technology, Trondheim, Norway; 3 Department of Rheumatology, St. Olavs University Hospital, Trondheim, Norway; 4 Department of Public Health and Nursing, NTNU – Norwegian University of Science and Technology, Trondheim, Norway; 5 Department of Immunology and Transfusion Medicine, St. Olavs University Hospital, Trondheim, Norway

**Keywords:** Body composition, Lifestyle, Physical activity, Psoriatic arthritis

## Abstract

**Introduction:**

Psoriatic arthritis (PsA) and low physical activity (PA) negatively influence body composition. We examined the relative strength of associations of PA and PsA with body composition in individuals with PsA compared to controls, and comparing PA assessment by self-report versus a device.

**Methods:**

We analyzed data from 142 individuals with PsA and 23 858 controls who participated in the fourth survey of the Trøndelag Health study (HUNT4, 2017–2019). Standardized multivariable linear regression models were used to assess the relative strength of associations of PsA (CASPAR criteria) and PA with visceral fat mass and percentage body fat measured by bioelectrical impedance analysis. PA was either self-reported (exercise frequency, duration and intensity) or device-measured using accelerometers. Model fit was compared using Akaike’s Information Criterion and the Bayesian Information Criterion.

**Results:**

Standardized regression coefficients for PsA had much smaller absolute values than those for performing PA in explaining variations in body composition (visceral fat mass: PsA: 0.017 to 0.019, PA: -0.14 to -0.31; percentage body fat: PsA 0.013 to 0.015, PA: -0.12 to -0.29). Unstandardized models showed that performing moderate to high levels of PA could compensate for the unfavorable body composition associated with PsA. Models with device-measured PA had substantially better fit than those with self-report, indicating that they better captured the variations in body composition.

**Conclusion:**

PA explained larger variations in body composition than PsA. Individuals with PsA should be informed that engaging in PA has a greater positive impact on body composition than the negative consequences of having PsA.

**Table:** 

**Key Points** *• Low physical activity was more strongly associated with an unfavourable body composition than psoriatic arthritis.* *• Device-measured physical activity more accurately captured the associations between physical activity and body composition than self-reported measures.* *• Promoting physical activity and perhaps use of wearable devices to record it may potentially help individuals with psoriatic arthritis mitigate the unfavorable body composition associated with their arthritis.*

## Introduction

Psoriatic arthritis (PsA) is a chronic inflammatory joint disease characterized by axial and peripheral arthritis, enthesitis, dactylitis, nail involvement, and skin psoriasis [1]. The prevalence of PsA is estimated to 0.01–0.67%, depending on the ethnic group and geographical locations [2]. The highest reported prevalence of PsA has been observed in central Norway [3]. Individuals with PsA have higher prevalences of other diseases, including hypertension, hyperlipidemia, type 2 diabetes, metabolic syndrome, and cardiovascular disease [4, 5]. Several studies have shown changes in body composition, especially higher visceral fat mass and percentage body fat among individuals with PsA [6, 7]. These changes in body composition contribute to a higher comorbidity burden [8, 9]. Therefore, understanding factors influencing the body composition may help enhance the management of comorbidities associated with PsA and improve patient outcomes.

PA has generally been associated with favorable health outcomes, including improved body composition and reduced metabolic risk factors [10]. Studies among individuals with PsA showed that higher levels of self-reported PA correlated with lower visceral fat mass and percentage body fat [11, 12]. However, individuals with PsA perform lower levels of PA compared to the general population [13, 14]. The relative contribution of PsA versus low PA to the unfavorable body composition remains unclear. This question is of clinical importance: if a PsA diagnosis in itself has a relatively greater influence on body composition, the potential benefits of increased PA would probably be smaller, and vice versa.

The aim of the present study was to examine the relative strength of the associations of PsA and PA with body composition, specifically visceral fat mass and percentage body fat, in individuals with PsA and controls using data from a large population-based study. We also aimed to compare these findings according to whether PA was assessed by a device or self-reported.

## Materials and methods

This is a cross-sectional study that utilized data from the fourth survey of the population-based Trøndelag Health study (HUNT4) in Norway [15]. In brief, HUNT includes surveys with questionnaires, clinical examinations, laboratory measurements, and biological sampling, with approximately 11-year intervals. In HUNT4 (2017–2019), all residents aged ≥ 20 years in the Northern part of Trøndelag were invited to participate.

## Study variables

### Psoriatic arthritis

To validate the diagnosis of PsA in our study, the medical records of participants with probable PsA in HUNT4 were reviewed by an experienced senior rheumatologist (MH) using the **ClAS**sification for **P**soriatic **AR**thritis (*CASPAR*) criteria [16].

### Physical activity

Self-reported PA in HUNT4 comprised three questions on frequency, duration, and intensity [17]. We converted the answers from the questionnaires to Metabolic Equivalent of Task hours per week (MET-hrs/week), similar to several previous studies [18–20]. First, for each included participant, we obtained weekly minutes spent on PA by multiplying frequency and duration of PA. Frequency of PA was assessed with the question “How often do you exercise (on average)?” Options were “Never,” “Less than once a week”, “Once a week”, “2–3 times per week” or “Nearly every day”, which were converted to 0, 0.5, 1, 2.5, and 5 weekly days, respectively. The duration of PA was assessed with the question “For how long do you exercise each time?” Options were “Less than 15 min”, “15–29 min” “30–60 min”, “More than 60 min”, which were converted to 7.5, 22.5, 45, and 60 min, respectively. Intensity of PA in HUNT4 was assessed with the question “How hard do you exercise?” with options “No sweat or heavy breath”, “Sweat or heavy breath”, and “Push myself to exhaustion”, which were converted to 3, 6 and 9 METs, respectively. We multiplied hours of PA per week with intensity to obtain a continuous variable of MET-hrs/week. The continuous variable was further categorized into low, moderate or high PA, based on World Health Organization (WHO) aerobic PA guidelines for healthy adults above 18 years, comprising at least 150–300 min of moderate-intensity aerobic PA or 75–150 min of vigorous-intensity aerobic PA pr week; or an equivalent combinations of these two [21]. This is approximately equivalent to low PA: ≤ 8.3 MET-hrs/week, moderate PA: 8.4–16.6 MET-hrs/week, and high PA: ≥ 16.6 MET-hrs/week, as also used elsewhere [20]. 1 MET-hr is defined as the ratio of work metabolic rate to a standard resting metabolic rate of energy expenditure of 1 kcal/kg body weight/hr (approximately 3.5 ml/O_2_/kg/min).

For device-measured PA, participants were asked to wear two tri-axial AX3 accelerometers (Axivity, Ltd., Newcastle, UK) for 7 consecutive days when attending the clinical examination in HUNT4. The AX3 is small and waterproof (dimensions: 23 × 32.5 × 7.6 mm; 11 g) with a 512 MB flash drive for off-line data storage. The OmGui software (version 1.0.0.43; Open Movement, Newcastle, UK) was used to configure, initialize, and download data before further processing.

The accelerometers were placed on the right thigh approximately 10 cm above the upper border of patella, and on the lower back on the third lumbar segment, respectively. To fix the sensors, a 5 × 7 cm adhesive film (Opsite Flexifix; Smith & Nephew, Watford, UK) was attached directly to the skin. The sensor was placed on top of the film using double-sided tape (3 M, St. Paul, MN, USA) and covered with a new 8 × 10 cm layer of adhesive film.

After completing the measurement, participants delivered the devices at the clinical examination site or sent them back in a pre-stamped envelope. The further processing of the data has been described in detail elsewhere [22]. In brief, the two raw data files from each participant were segmented into 5 s windows (250 samples) before computing 161 different time- and frequency features for each window. These features were then fed into a machine learning model trained to predict lying down, sitting, standing, slow walking (≤ 4 km/hr), moderate walking (4.1–5.4 km/hr), brisk walking (≥ 5.5 km/hr), running, and cycling [23, 24]. An additional machine learning model was trained to detect no-wear time [25]. If no-wear time was predicted for at least one hour/day, the entire 24 h was excluded. The first and last days of measurements were also excluded (i.e., the days with mounting and taking off the accelerometers). Therefore, only days with complete 24 h accelerometer recordings were included in the analyses.

The amount of device-measured PA was quantified as MET-hrs/week by multiplying the estimated MET value of each PA type (i.e., slow walking (≤ 4 km/hr): 2.8 MET, moderate walking (4.1–5.4 km/hr): 3.5 MET, brisk walking (≥ 5.5 km/hr): 4.5 MET, bicycling: 5.8 MET, and running (> 6.4 km/h): 6.5 MET) by the hours spent for each PA type per day. Sitting and standing postures were not included. The MET values for each of the PA types were based on the Ainsworth compendium of physical activity [26]. We further categorized the continuous variable into tertiles, defining the lowest tertile as low-level PA, the middle as moderate, and the highest as high-level PA. This was done because categorization of device-measured PA, which captures total daily PA, was not directly comparable to self-reported PA, which primarily focuses on exercise. To our knowledge, there is no established recommendation for categorizations of total PA.

### Dependent variables

Visceral fat mass and percentage body fat were the dependent variables in our study. They were measured with bioelectrical impedance at the clinical examination in HUNT4 (InBody 770, Cerritos, CA, USA) [15].

### Inclusion and exclusion criteria

**Fig. 1:**
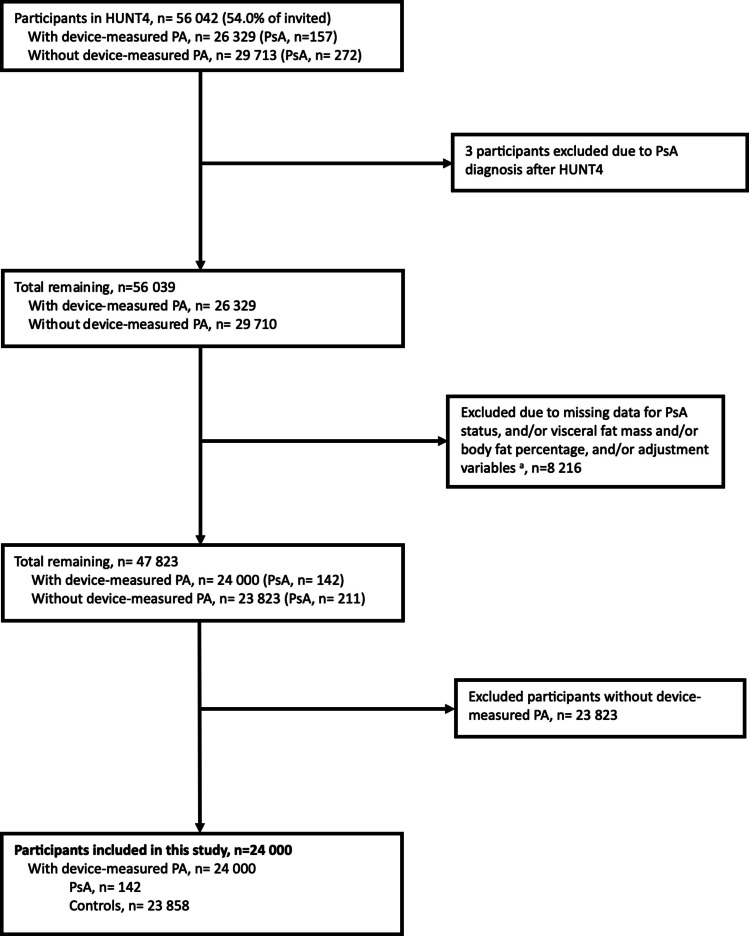
Participant inclusion and exclusion to the study. ^a^Missing data for self-reported PA *n* = 14 (0.02%) and adjustment variables including sex, *n* = 0, smoking, *n* = 275 (0.5%), lung diseases, *n* = 1728 (3.1%), and heart disease, *n* = 1859 (3.3%), height, *n* = 0, and month of participation in HUNT4, *n* = 0. Abbreviation: HUNT4: The fourth survey of the Trøndelag Health study (2017–2019). PsA: Psoriatic arthritis. PA: Physical activity

Figure 1 shows the inclusion to the study. The main inclusion criterion was participation in HUNT4. We excluded participants with missing data on PsA status, a PsA diagnosis after HUNT4, or missing data for visceral fat mass, percentage body fat, self-reported PA, or adjustment variables as detailed in Fig. 1. After exclusions, the study included 142 individuals with PsA and 23 858 controls.

### Statistics

Statistical analyses were performed using Stata (16.1 StataCorp. College Station, TX, USA). *p*-values < 0.05 were considered statistically significant. Data are given as means with 95% confidence intervals (CI) or number with percentages. Normal distribution was evaluated with histograms. We compared background characteristics of individuals with PsA and controls using Pearson’s chi-square test for categorical variables, and Student’s t-test or the Mann–Whitney U test for continuous variables.

To investigate the study aim, multivariable linear regression modeling was performed with visceral fat mass or percentage body fat as dependent variables, and PsA and PA as the independent/study variables. The models were adjusted for variables influencing both PA and body composition, available in HUNT4: age, sex, smoking status (present, former, or never smoker), lung disease (yes/no, yes = chronic obstructive lung disease or asthma), and heart disease (yes/no, yes = myocardial infarction or heart failure). We also adjusted for height to account for differences in body size and for the month of participation to adjust for seasonal variations in PA [27]. As detailed above, alternative measurement forms for PA included categorized self-reported PA (low/moderate/high MET-hrs/week), as well as similarly categorized or continuous device-measured PA (MET-hrs/week). All analyses were conducted using the same sets of participants and adjustment variables. Standardized regression coefficients were used to compare the relative strength of associations of the study variables to explain variations in visceral fat mass and percentage body fat. In standardized models, all variables were expressed in standard deviation (SD) units, enabling direct comparison of regression coefficients. This direct comparability would not be possible when, for example PsA was measured as a yes/no variable and PA was measured in MET-hrs/week. To investigate whether associations between PA and body composition differed between individuals with PsA and controls, an interaction term between PsA and PA was tested in each model. We also tested models including diabetes (yes/no) to investigate whether this factor influenced the association between PA and body composition.

To evaluate model fit, we used* R*-squared, Akaike´s Information Criterion (AIC) and Bayesian Information Criterion (BIC). AIC and BIC are penalized-likelihood information criteria widely used to select the best-fitting model among alternative models including the same participants [28]. Lower values suggest better model fit; a role of thumb is that 2 units difference of AIC or BIC (Δ AIC or Δ BIC = 2) is regarded as moderate evidence and a difference of 4 units as strong evidence [29]. However, AIC and BIC values themselves depend on the number of participants and the complexity of each model.

To illustrate the degree of subjectivity of self-reported PA, histograms showing device-measured PA (continuous MET-hrs/week) in participants with or without PsA within each category of self-reported PA were used. To study whether these data were different in individuals with PsA compared to controls Student’s t-test was used, comparing mean MET-hrs/week between individuals with PsA and controls within each category.

### Ethics

Participants in HUNT gave written informed consent. The present study complied with the Declaration of Helsinki and was approved by the Regional Committee for Medical and Health Research Ethics, Northern Norway (#11,926).

## Results

Participant characteristics are given in Table 1. The study included 142 individuals with PsA and 23 858 controls with complete data for relevant variables. Background characteristics showed that individuals with PsA were older, more often smokers, had higher rates of unemployment, and higher prevalences of diabetes and hypertension. (Table 1). They also had more unfavorable body composition, indicated by higher weight, body mass index, waist-hip ratio, visceral fat mass, and percentage body fat as shown in Table 2.

**Table 1:** Participant characteristics

Variable	PsA, *n* = 142	Controls, *n* = 23 858	*p*-value
Age years, mean (SD)	58 (11)	53 (16)	*p* < 0.001
Years with PsA diagnosis at time of study participation			
9 years or less, *n* (%)	93 (65)	-	
10–19 years, *n* (%)	32 (23)	-	
20 years and above, *n* (%)	17 (12)	-	
Male, *n* (%)	84 (59)	13 423 (56)	*p* = 0.49
Diabetes^a^, *n* (%)	15 (11)	1 117 (5)	*p* = 0.001
Heart disease^b^, *n* (%)	7 (5)	828 (3)	*p* = 0.34
Hypertension^c^, *n* (%)	71 (50)	8 327 (35)	*p* < 0.001
Respiratory disease^d^, *n* (%)	20 (14)	2 988 (13)	*p* = 0.58
Hypercholesterolemia, *n* (%)	29 (21)	4 759 (20)	*p* = 0.89
Cancer, *n* (%)	15 (11)	1 704 (7)	*p* = 0.12
Serum triglycerides mmol/L, mean (SD)	1.9 (1.2)	1.6 (1.0)	*p* < 0.001
HDL cholesterol mmol/L, mean (SD)	1.4 (0.4)	1.4 (0.4)	*p* = 0.86
Systolic blood pressure mmHg, mean (SD)	131 (18)	128 (18)	*p* = 0.04
Diastolic blood pressure, mmHg, mean (SD)	75 (9)	73 (10)	*p* = 0.02
CRP mg/L, mean (SD)	3.4 (7.0)	2.4 (4.8)	*p* = 0.011
eGFR ml/min/1.73m^2^, mean (SD)	90 (15)	93 (18)	*p* = 0.14
Alcohol (twice a week or more), *n* (%)	33 (23)	5 149 (22)	*p* = 0.65
Smoking status, n (%)			*p* = 0.001
Present	17 (12)	2 032 (9)	
Former	83 (58)	10 895 (45)	
Never	42 (30)	10 931 (46)	
Education (approved apprenticeship, college or higher), *n* (%)	83 (58)	15 688 (66)	*p* = 0.062
Unemployed at the time of HUNT4 participation, *n* (%)	62 (44)	8 050 (34)	*p* = 0.013

^a^Diabetes: self-reported diabetes and/or the use of anti-diabetic medication and/or having a non-fasting blood-glucose level > 11 mmol×L^−1^

^b^Heart disease: Self-reported myocardial infarction and/or heart failure

^c^Hypertension: Systolic blood pressure≥140 mm Hg and/or a diastolic blood pressure≥90 mm Hg and/or self-reported use of antihypertensive medication

^d^Respiratory disease: Self-reported asthma and/or chronic obstructive pulmonary disease

*HDL* High-density lipoprotein. *CRP* C-reactive protein. *eGFR* estimated glomerular filtration rate. *HUNT4* The fourth survey of the Trøndelag Health study (2017-2019)

**Table 2:** Physical activity and body composition

Variable	PsA, *n* = 142	Controls, *n* = 23 858	*p*-value
Height cm, mean (SD)	170 (8)	171 (9)	*p* = 0.086
Weight kg, mean (SD)	83 (15)	79 (16)	*p* = 0.020
Body mass index kg/m^2^ (SD)	28.5 (4.4)	26.9 (4.5)	*p* < 0.001
Waist-hip ratio, mean (SD)	0.98 (0.08)	0.95 (0.08)	*p* < 0.001
Body fat mass kg, mean (SD)	28.2 (9.8)	24.6 (10.3)	*p* < 0.001
Visceral fat mass kg, mean (SD)	13.4 (5.3)	11.4 (5.4)	*p* < 0.001
Percentage body fat, mean (SD)	33.7 (8.4)	30.5 (9.2)	*p* < 0.001
Physical activity level^a^, n (%)			*p *= 0.87
Low	70 (49)	11 277 (47)	
Moderate	48 (34)	8 242 (35)	
High	24 (17)	4 339 (18)	

^a^Based on categorized MET-hrs/week calculated from self-reported physical activity

The regression models confirmed that PsA was significantly positively associated with visceral fat mass and percentage body fat, whereas moderate and high self-reported or device-measured PA was significantly negatively associated with the dependent variables (Table 3). The standardized regression coefficients for PsA ranged from 0.017 to 0.019 in the models for visceral fat mass, whereas the absolute values of the coefficients for PA were much larger, ranging from −0.14 to −0.31. Similarly, the standardized coefficients for PsA in the models for percentage body fat ranged from 0.013 to 0.015 and those for PA ranged from −0.12 to −0.29. This indicates that PA explained a lot more of the variations in the dependent variables than PsA. The coefficients from the non-standardized models confirmed this finding, showing that individuals with PsA performing moderate or high levels of PA on average had larger visceral fat mass and percentage body fat reductions than the increased amounts associated with their arthritis. The interaction term between PsA and PA was not statistically significant (*p*-values ranging from 0.27 to 0.94) in any of the models, indicating that the associations of study variables with body composition were similar for individuals with PsA and controls. Including diabetes in the models did not change the associations between PA and body composition but led to reduced model fit.

**Table 3:** Associations of psoriatic arthritis and physical activity with visceral fat mass and percentage body fat

Primary outcome	PA measurement form	Study variables	Regression coefficient (95% confidence interval)	Standardized coefficient	R-squared	ΔΔ AIC	ΔΔ BIC
Visceral fat mass	Self-reported PA	PsA	1.4 (0.5, 2.2) *	**0.019**			
		MET-hrs/week^a^			0.16	Reference	Reference
		Low	Reference				
		Moderate	-1.6 (-1.5, -1.8) **	**-0.14**			
		High	-3.2 (-3.0, -3.4) **	**-0.23**			
	Devicemeasured PA	PsA	1.3 (0.5, 2.1) *	**0.018**			
		MET-hrs/week^b^			0.19	- 776	- 774
		Low	Reference				
		Moderate	-2.2 (-2.0, -2.3) **	**- 0.19**			
		High	-3.8 (-3.6, -3.9) **	**- 0.33**			
		PsA	1.2 (0.4, 2.0) *	**0.017**			
					0.19	- 1 021	- 1 029
		MET-hrs/week (continuous)	-0.1 (-0.1, -0.1) **	**- 0.31**			
Percentage body fat	Self-reported PA	PsA	1.8 (0.6, 3.0) *	**0.015**			
		MET-hrs/week^a^			0.39	Reference	Reference
		Low	Reference				
		Moderate	-2.3 (-2.1, -2.5) **	**- 0.12**			
		High	-5.0 (-4.7, -5.2) **	**- 0.21**			
	Devicemeasured PA	PsA	1.6 (0.4, 2.8) *	**0.014**			
		MET-hrs/week^b^			0.40	- 664	- 664
		Low	Reference				
		Moderate	-3.0 (-2.8, -3.2) **	**- 0.15**			
		High	-5.6 (-5.3, -5.8) **	**- 0.29**			
		PsA	1.6 (0.4, 2.8) *	**0.013**			
					0.41	- 1 017	- 1 025
		MET-hrs/week (continuous)	- 0.2 (-0.2, -0.2)**	**- 0.27**			

^a^Self-reported frequency, duration, and intensity of physical activity converted to MET-hrs/week, and categorized to Low PA: ≤ 8.3 MET-hrs/week, Moderate PA: (8.4–16.6 MET-hrs/week), and High PA: ≥ 16.6 MET-hrs/week

^b^Accelerometer-measured MET-hrs/week were categorized into tertiles; low, moderate, and high PA

*MET-hrs/week* Metabolic Equivalent of Task hours per week, *PA* Physical activity, *AIC* Akaike´s Information Criterion *BIC* Bayesian Information Criterion

*< 0.05

**< 0.001

Table 3 also shows that the relative strength of associations of PsA and PA on the associations with body composition were similar whether PA was self-reported or device-measured. However, the models with device-measured PA had significantly better fit compared to models with self-reported PA, evident by the substantially lower values of AIC and BIC in models with device-measured PA (Table 3). This indicates that device-measured PA better captured the variations in body composition than did self-reported PA. Both for visceral fat mass and for percentage body fat, the models with continuous device-measured PA (i.e. continuous MET-hrs/week) had the best fit.

**Fig. 2:**
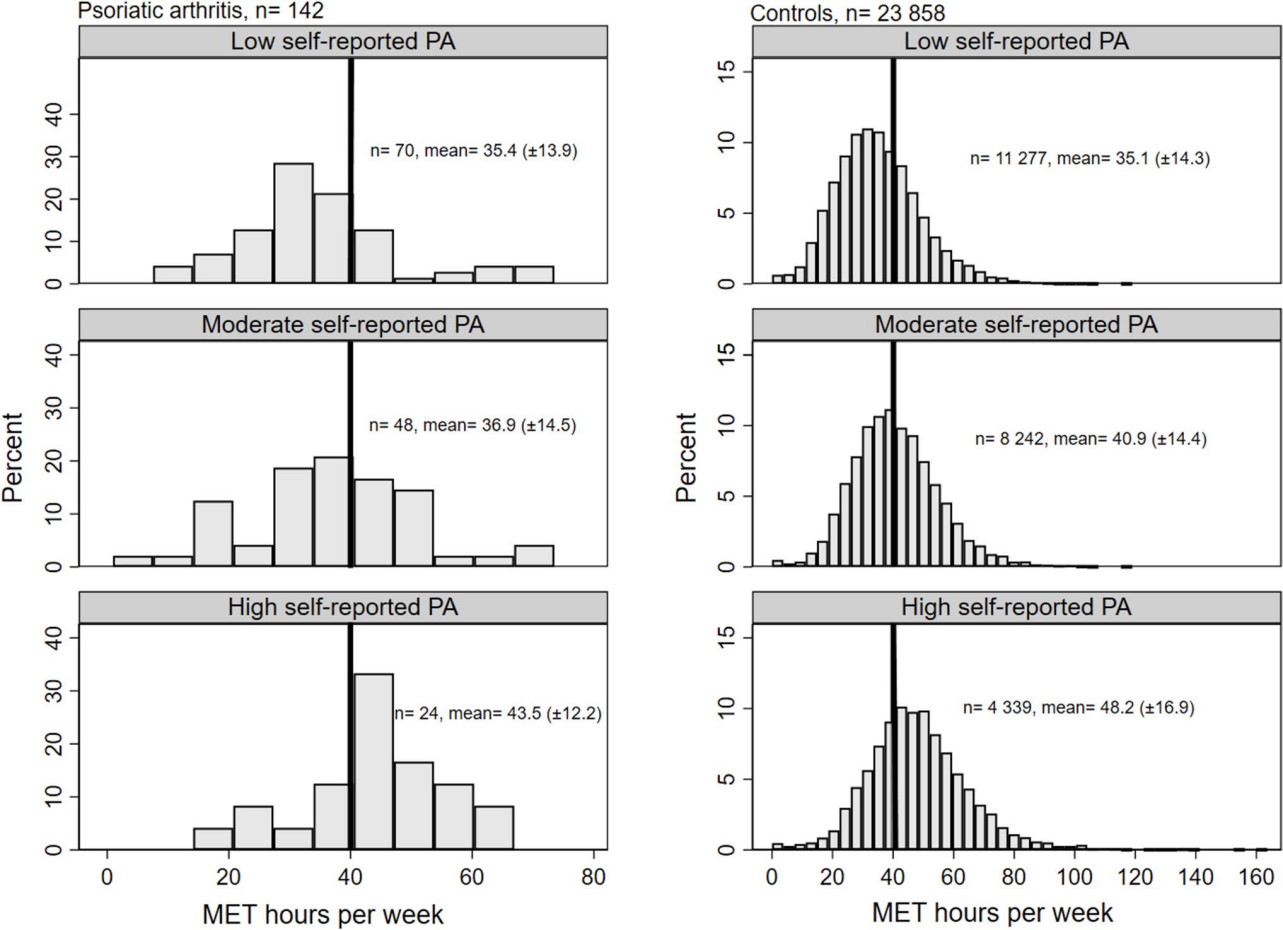
Accelerometer-measured MET-hrs/week in participants with different levels of self-reported PA. The vertical line is a reference line at 40 MET-hrs/week to ease visual comparisons. The distributions of the continuous accelerometer-measured PA variable (MET-hrs/week) shifts to the right, showing higher values of MET-hrs/week) within higher levels of self-reported PA. Note the large variations within each PA category. Abbreviation: MET: Metabolic Equivalent of Task. PA: Physical activity

The histogram showed a wide variation in the continuous device-measured PA variable (i.e., continuous MET-hrs/week) within each level of self-reported PA both for individuals with PsA and controls, even if the mean of the continuous variable increased with higher levels of self-reported PA (Fig. 2). This illustrates that for a given level of self-reported PA, some individuals substantially overreport and others substantially underreport their PA if the device measurements from one week of registration are used as “gold standard”. However, there were no statistically significant differences in mean MET-hrs/week between individuals with PsA and controls within each group of self-reported PA (Low PA: *p* = 0.86, Moderate PA: *p* = 0.053, High PA: *p* = 0.16).

## Discussion

As expected, both PsA and PA were significantly associated with body composition. However, the present study showed that PA was more strongly associated with visceral fat mass and percentage body fat than PsA. Furthermore, the associations between PA and body composition were similar among individuals with PsA and controls. The results were also similar whether PA was self-reported or device-measured, but device-measured PA better captured the associations as shown by much better model fit.

Previous research has shown that having a diagnosis of PsA is associated with an unfavorable body composition [6, 7], whereas PA consistently has been associated with favorable body composition outcomes [11, 12]. To our knowledge, the relative association of PsA vs PA on body composition among individuals with PsA has not previously been examined. The present study demonstrated that PA had stronger associations with body composition than the diagnosis of PsA itself. The relationship between PA and body composition may be bidirectional. However, in light of a previous study demonstrating a causal relationship between PA and changes in body composition among individuals with PsA [11], our findings suggest that higher PA levels could contribute to mitigating some of the unfavorable body composition profile associated with PsA. This is of clinical importance because PA is a modifiable lifestyle factor that clinicians can target when caring for individuals with PsA. Motivation for performance of PA may be strengthened by knowing that regardless of the presence of PsA, higher levels of PA are associated with a meaningfully improved body composition. In turn, achieving PA and body compositions goals could reinforce the value of active self-management for better health outcomes.

The study also demonstrated that PA had a similar association with body composition among individuals with PsA and controls. This supports that individuals with PsA can achieve body composition benefits from PA that are comparable to those observed in the general population. Taken together, our findings add further support to the recommendation that health care providers should integrate structured PA counselling and lifestyle interventions into routine PsA management alongside pharmacological treatment [30].

Our study also showed similar relative associations of PsA and PA with body composition when PA was self-reported and device-measured. However, device-measured PA more accurately captured the associations with body composition, as reflected by better fitting models. Given that individuals with PsA are less physically active than the general population [13, 14] and that even small increases in PA have health benefits [31], precise estimation of PA levels may be particularly important among individuals with inflammatory arthritis, including PsA.

The superiority of device-measured PA to better estimate the variations in body composition could be because it captured total weekly PA, including both planned and incidental activities during work and leisure-time [32]. This supports that total PA plays an important role for body composition [33]. In contrast, self-reported PA in our data focuses primarily on exercise (frequency, duration and intensity). Another key distinction between the two measurement forms is that while device-measured PA records data over a short timeframe, i.e. one week in our study, self-reported PA often reflects the habitual exercise over a longer period.

Self-reported PA relies on recall and subjective perception, which can lead to overestimation [34] particularly in individuals with joint pain, and underestimation of unstructured activities not perceived as exercise [34]. Despite these limitations, self-reported PA remains convenient and cost-effective, and therefore has been widely used in large epidemiological studies. On the other hand, device measurements are resource-demanding in large studies as they require specialized equipment and skills for data interpretation [35]. Moreover, there are no standardized methods for data collection and processing. Consequently, incorporating device-measured PA may not always be feasible in large epidemiological studies.

Our study has several strengths. PsA was validated based on the CASPAR criteria by an experienced rheumatologist, which enhances the reliability of our findings. Additionally, we utilized device-measured PA and assessed body composition through bioelectrical impedance analysis, further strengthening the robustness of our results. The sample size was large, which also permitted meaningful comparisons between PA measurement methods. However, there are limitations to consider. The cross-sectional design of the study does not allow for determination of the nature of the associations between PA and body composition, and the relatively small number of individuals with PsA reduced the statistical power. We also did not have data on the nutritional status of the participants in our study, which is known to influence body composition [36]. The MET values used to assess PA were primarily based on data from young adults, which may overestimate the PA levels of older individuals [26].

## Conclusion

Our study showed that PA could explain much larger variations in the body composition than PsA among individuals with PsA. Since PA is a modifiable factor, individuals with PsA should be encouraged to engage in WHO-recommended levels of PA, which could outweigh some of the negative effects of their arthritis and help them achieve a more favorable body composition. Further research is needed to explore the long-term effects of PA on body composition, its role in managing comorbidities, and its potential to improve overall health outcomes in individuals with PsA.

## Data Availability

Data from HUNT are available upon reasonable request from the HUNT Research Centre (www.ntnu.edu/hunt/data), following approval from the Regional Ethics Committee. However, restrictions apply to the availability of the data for the present paper, which were used under license for the current study and are not publicly available in accordance with Norwegian law.
